# Effects of Regular Taekwondo Intervention on Health-Related Physical Fitness, Cardiovascular Disease Risk Factors and Epicardial Adipose Tissue in Elderly Women with Hypertension

**DOI:** 10.3390/ijerph18062935

**Published:** 2021-03-12

**Authors:** Yun Hwan Kim, Min Ki Jeong, Hyuntae Park, Sang Kab Park

**Affiliations:** 1 College of Arts and Sports, Dong-A University, Busan 49315, Korea; ynani3721@naver.com (Y.H.K.); prof.mkjeong@gmail.com (M.K.J.); 2 Department of Health Sciences, Graduate School, Dong-A University, Busan 49315, Korea; 3 Institute of Convergence Bio-Health, Dong-A University, Busan 49315, Korea

**Keywords:** taekwondo, health-related physical fitness, cardiovascular disease risk factor, epicardial adipose tissue, hypertension

## Abstract

Regular exercise has been proven to prevent hypertension and to help in the management of hypertension. There is a lack of studies examining changes in these issues as a result of Taekwondo training intervention. The aim of the current trial is to identify the effects of a regular Taekwondo (TKD) training program on health-related physical fitness (HRPF), cardiovascular disease (CVD) risk factors, inflammatory factors, and epicardial adipose tissue (EAT) in elderly women with hypertension. To accomplish this, 20 participants, who were older women with hypertension, were divided into a TKD group (*n* = 10) and a control group (*n* = 10). The TKD program was conducted in program for 90 min, three times a week, for 12 weeks. Outcomes, including body composition, blood pressure (BP), HRPF, cardiovascular risk factor and EAT, were measured before and after the Taekwondo program. The 12-week TKD program improved body composition, BP, HRPF, CVD risk factor, and EAT in elderly women with hypertension relative to controls. Meanwhile, EAT and interukin-1β (r = 0.530, *p* < 0.05), monocyte chemotactic protein-1 (r = 0.524, *p* < 0.05), triglyceride (r = 0.493, *p* < 0.05) and sedentary behavior (r = 0.459, *p* < 0.05) presented a positive correlation, while EAT and lean body mass (r = −0.453, *p* < 0.05) showed a negative correlation. The 12-week regular TKD training intervention was found to be effective in reducing the thickness of EAT measured by multi-detector computed tomography and can also enhance health-related physical fitness and risk factors of CVD in older individuals with hypertension.

## 1. Introduction

Hypertension causes the regeneration of blood vessels inside blood vessels, increases shrinkage and the activation of cell proliferation of smooth muscle [1], and is reported to be a high-risk factor of cardiovascular disease (CVD) [2].

According to the report from Statistics Korea [3], the number of deaths of elderly women owing to cerebrovascular disease occupies the first rank requiring preventive measures. Major causes of the onset of CVD comprise diverse ones including obesity owing to non-physical activity (PA), heredity, smoking, gender, age, family history of type 2 diabetes mellitus, and hypertension, etc. [4,5,6,7]; the obesity due to an increase in adipose tissue is also an important risk factor [8,9,10] causing metabolic diseases such as CVD, type 2 diabetes mellitus, and hyperlipidemia.

Taguchi et al. [11] reported that pericardial fat is also a risk factor of coronary artery disease as well as abdominal fat. More specifically, he suggested that the higher occasions of the onset of CVD would be probable in case of the abundance of pericardial fat though the level of visceral fat was low. The Epicardial Adipose Tissue (EAT), which is a form of adipose tissue, is attached to the heart directly without fascia, thereby influencing the pathophysiological function of the heart directly, and is employed as a favorable indicator to predict CVD compared to other adipose tissue [12,13,14,15].

According to recent research, the EAT secretes several kinds of hormone to allow visceral adipose tissue to play the role of metabolic active endocrine organs and is reported as the cause of an inflammatory mediator affecting CVD and coronary atherosclerosis with regard to its location adjacent to myocardium and coronary artery [16,17,18].

Also, the sustainment of an overload of fatty acid over EAT would result in the secretion of inflammation inducing the cytokine and cell growth factor of smooth muscle from fat cells around the heart to adjacent coronary artery [19]; Mazurek et al. [20] reported EAT as one of origins of inflammatory cytokines with high measurements of Chemokine (Monocyte Chemotactic Protein-1; MCP-1) and inflammatory mediator (Interleukin-1β; IL-1ß, Interleukin-6; IL-6, Interleukin-6 soluble receptor; IL-6 soluble receptor, MCP, Tumor necrosis factor-α; TNF-α) from EAT [21].

Deficiency in PA is a major cause of the onset of CVD [22], and a sedentary life sitting for one hour everyday would increase the level of fat in the heart by 2.39 cm^2^ [23]. In particular, exercise is deeply related to EAT in that EAT has an even higher relevance to whole body endurance compared to other adipose tissue in the human body [24]. Thus, regular exercise has been reported to decrease the level of EAT, blood pressure (BP), and death rate ascribable to CVD [25,26].

Previous studies, which delved into the relationship between physical exercise and CVD risk factor and EAT, rendered the following results. Palmefors et al. [27] reported the positive effect of exercise upon vascular adhesion molecule (vascular cell adhesion molecule-1; VCAM-1, Intercellular Adhesion Molecule-1; ICAM-1) and inflammatory factors (Tumor necrosis factor-α; TNF-α, C-reactive protein; CRP) in the research on CVD risk factor, PA, and exercise. Park et al. [28] showed significant improvement in CVD risk factor (total cholesterol: TC, high-density lipoprotein cholesterol: HDL-C, Insulin; all: *p* < 0.05) and a decrease in EAT thickness (20%) upon execution of a swimming program for 12 weeks for obese adult male (35.5 ± 4.84, *n* = 10). Additionally, the execution of aerobic exercise (60–70% heart rate max: HRmax, 60 min/day, 3 days/wk) which lasted for 12 weeks for obese men left a significant decrease in EAT thickness [29].

Taekwondo (TKD), a form of sports and martial arts that originated in South Korea, is a popular sport with currently about 80 million members in 200 countries widely distributed around the world, a number that continues to grow [30]. In particular, in South Korea, it is very familiar and can be practiced anywhere around the house, while a large number of people practice it, so it has excellent accessibility. In addition, TKD has been appreciated as an integrated exercise comprising diverse basic movements improving physical strength for health and motor function [31] in which the special movement of body improves PA [32], and Bridge et al. [33] reported that the exercise intensity of TKD can be adjustable depending on the type of TKD training and the degree of application. According to previous studies, Taekwondo is helpful in improving health for the elderly, and, in particular, positive reports related to falls and increased muscle strength have been published [34,35].

However, although the kinesiological approach is important because hypertension is a high-risk factor for CVD in the elderly, there are few TKD intervention studies to mediate hypertension in the elderly population. Also, there is only very limited evidence on the effect of TKD in elderly women with hypertension, and it is still unclear how exactly TKD intervention induces CVD risk factor and EAT in the hypertension.

In the present research, the TKD program comprising TKD aerobics using music and the basic movement of TKD (stance, punch, and kick) was designed by referring to recommendations for elderly people with hypertension presented in the American College of Sports Medicine (ACSM) [36] for the application of the program to elderly women with hypertension to demonstrate the effect of improvement in BP and CVD risk factor through TKD program intervention.

Thus, the present study intends to identify the effects of the TKD program on health-related physical fitness (HRPF), CVD risk factor, inflammatory factors, and the EAT of elderly women with hypertension. In addition, it is intended to investigate the relationship between EAT and physical capacity, lipid profile and inflammatory variables.

## 2. Materials and Methods

### 2.1. Ethical Approval and Determination of Study Size

This study was approved by the institutional review board (IRB) of Dong-A University, Busan, South Korea (2-1040709-AB-N-01-201904-HR-017-06), and was conducted in accordance with the ethical standards of the declaration of Helsinki. All participants were told about the purpose of the study and the study design, and all participants signed written informed consents.

To determine the sample size, the G*Power version 3.1.9.2 statistical power analysis software program was used. Based on data from a pilot study, the estimated sample size required to obtain a minimum power of 80% at a significant alpha of 95% was 10. According to a prior pilot test, at least 12 participants were required in each of the two study groups. Accordingly, 12 participants were recruited to account for a potential dropout rate of 20%. The alpha level was 0.05.

### 2.2. Participants

**Figure 1:**
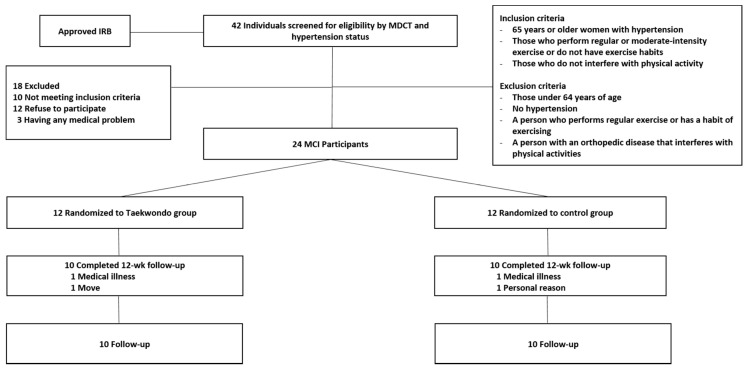
Flow diagram of participants and follow-up.

A single-blinded, randomized controlled trial was conducted. The twenty-four elderly women with hypertension subjects of this study were attached using the bulletin board of the several welfare centers managed by Sahagu Sarang-chae Elderly Welfare Center in Busan, South Korea, or recruited applicants through the [public announcement of recruitment of participants] on the website. By numbering the names of those who agreed to the study in order, a computer program (https://www.randomizer.org (accessed on 22 April 2019) was used to randomly assign 24 subjects to the TKD (*n* = 12) group and control groups (*n* = 12). Four of them were excluded from the study for personal reasons. The final study sample comprised a TKD group and a control group (*n* = 10 each), 20 subjects in total (Figure 1). The participants’ physical characteristics are shown in Table 1. The hypertension diagnosis was based on medical evaluations through a clinical interview by a heart specialist (Cardiologist). The diagnosis was also based on Multi-Detector Computed Tomography (MDCT) assessments. The presence of ischemic CVD such as EAT thickness and coronary artery stenosis calcification, etc., was identified, and the elderly women of 65 years old or older with BP medication from diagnosed hypertension (systolic BP (SBP) 140~159 mmHg or diastolic BP (DBP) 90~99 mmHg), among those medically granted to practice exercise, were selected as participants in the present study. Meanwhile, the control group gave them the opportunity to participate in the movement after all the experiments were completed. In addition, as a sign of gratitude for participation in the study, lunchboxes and snacks after the pre-test and post-test, sportswear after the pre-test, and a gift certificate after the post-test were provided.

### 2.3. Measurement of Body Composition and BP

Physical and anthropometric variables were measured at before the intervention and after 12 weeks in both groups. The body height, body weight, lean body mass (LBM), and percent body fat were measured using the Venus 5.5 body composition analyzer (Jawon medical Co., Kyungsan-City, Korea). Body mass index (BMI) was calculated as weight (kg) divided by height squared (m^2^), a tapeline (Martine’s body measuring instrument) was used to measure waist circumference (WC) and hip circumference (HC), and waist hip ratio (WHR) was calculated with a waist/hip ratio formula.

For BP, the study measured systolic BP (SBP) and diastolic BP (DBP) twice in resting and took the average value by using the mercury-free BP monitor (CK-E301, Chin Kow Medical Instrument Co., Ltd., Taipei, Taiwan). In addition, pulse pressure (PP) was calculated as SBP-DBP, and mean arterial pressure (MAP) was calculated as [MAP = DBP + ((SBP-DBP)/3)].

Also, myocardial oxygen consumption rest (MVO_2_ rest) was calculated as (RHR × SBP) ÷ 1000 using RHR and SBP [37].

### 2.4. PA and Sedentary Behavior Measurement

Details of the procedure have been described previously [38,39]. In brief, an accelerometer (Kenz lifecoder, Tokyo, Japan) was attached to a waist belt on either the left or right side of the body. This device measured the number of step counts and the intensity of PA and sedentary behavior every 4 s throughout each day for 12 weeks. The metrics of PA measured intensity (metabolic equivalents: METs) [40] and the daily step count (step/day) [41] of PA (moderate to vigorous PA, MVPA) from moderate-intensity (moderate-intensity effort 3–6 METs) to high-intensity (high-intensity effort >6 METS) except for low-intensity (low-intensity effort <3 METs). As for the sedentary behavior measurement criteria, all sedentary behaviors, such as sleep time, screen time, reading and driving, were analyzed except MVPA [42].

### 2.5. HRPF Measurement

According to the ACSM guidelines to HRPF, muscular strength, cardiorespiratory endurance, and flexibility are the factors defining the healthy status of physical strength [43]. For the muscular strength test, hand grip strength and repeated chair stand were tested; for flexibility, trunk flexion in a sitting position (TFS) was tested; for cardiorespiratory endurance, 2 min walking was conducted.

### 2.6. EAT Thickness Measurement

The 64-channel MDCT (GE Light speed VCT System) was used for the measurement of EAT for which the contrast media (OMNIPAQUEtm 350 mg I/mL GE Healthcare) were provided through intravenous injection. Collimation of 64 × 0.625 mm, slice thickness of 1.25 mm, pitch of 0.16, rotation time of 0.35, 100 kvp, and 450 ms were set as scan parameters, and the visual image was captured in accordance with synchronized electrocardiogram(ECG) with the use of 20 G needle. In total, 75 cc of contrast media was injected into brachial veins at the rate of 4.5 cc per second, and the ‘Smart Prep’ was set on pulmonary artery to obtain images 13 s after the point of time of HU 150. Images of a cross-section from the pulmonary arterial duct to the base of heart were obtained while breathing was stopped. When HR exceeds 60 times, the Indenol 20 mg was provided through oral administration and the HR was controlled after 1 h followed by subsequent examination; the NGT 0.6 mg was injected to the sublingual gland and examination was taken before capturing images through Computed Tomography (CT).

The method of measurement of EAT mass is as follows. First, the volume around the heart was extracted through manual tracking of the visceral layer of the envelope of the heart and the graph, and the area of interest of the x-section, wherein the value of the threshold was set, is depicted, and then the axial- and coronal images are captured from screen. Second, the two dotted lines appear in the graph, where the left and right dotted lines represent values of the minimum and maximum, respectively. Third, the 3D image of the heart was then made, from which the thickness of specific density (−200 ≤ CT ≤ −50 Hounsfield Units) of cardiac CT was calculated.

### 2.7. TKD Program

For the implementation of the TKD program, the recommendations for patients with hypertension presented in the ACSM guidelines [43] were referred to [44,45,46], and the TKD program of Jeong et al. [47] was modified and supplemented for the warm-up and cool-down for 10 min and the main exercise for 70 min; the program was implemented by 90 min for each session, three times a week, and lasted for 12 weeks. A period of 1~4 weeks of TKD program consisted of basic movement, whereas the period of 5~12 weeks comprised exercises enabling active movement with music. The TKD program was held every week (Monday, Wednesday, and Friday) at 10:30 am to 12:00 am in the sports room of the Sahagu Sarang-chae Elderly Welfare Center. Taekwondo education was conducted by two taekwondo leaders (license holders) with the participation of research staff with a doctorate in physical education. The control group was required to maintain their daily life.

During exercise, heart rate was measured by wearing the portable wireless heart rate meter POLAR (RA-400™, Kempele, Finland). The set-up for exercise intensity was 40~59% of HRR in Week 1~4 and 60~75% in Week 5~12. The TKD programs are shown in Table 2.

### 2.8. Blood Analysis

The participants’ blood samples were collected between 9 am and 10 am baseline and after the intervention. Eight-hour fasting prior to testing and limiting intense PA during 24 h was recommended to the subjects. Blood in the amount of 10 mL was collected from the forearm with a cannula, using a disposable syringe (Bom Medrea Co., Ltd.). Blood samples were centrifuged at 3000 rpm for 10 min and stored at −80 °C. The concentrations of serum TC, triglyceride (TG), low-density lipoprotein cholesterol (LDL-C), and HDL-C were analyzed using an automatic chemical analyzer (Hitachi-7600-110/7170 analyzer, Tokyo, Japan). Serum IL-1β, TNF-α, high-sensitivity CRP (hs-CRP), MCP-1, soluble VCAM-1 (sVCAM-1), soluble ICAM-1 (sICAM-1), and soluble E-selectin (sE-selectin) were determined enzymatically (Enzyme-Linked Immunosorbent Assay, ELISA) using standard laboratory procedures.

### 2.9. Statistical Analysis

Statistical analysis was performed using SPSS version 23.0 for Windows (IBM, Chicago, IL, USA). The descriptive statistics of all variables were calculated as means (M) and standard deviations (SD). The Shapiro–Wilk test for normality was used to confirm the normal distribution of all outcome variables. The unpaired participations *t*-test was used to assess group differences in baseline (beginning of the intervention) variables. A two-way repeated ANOVA was used to examine interactions between period and group and differences between groups. In the case of a significant period by group interactions, a paired *t*-test was performed to examine differences between baseline and 12 weeks. Pearson’s correlation coefficients (r) were calculated to identify associations between EAT and physical capacity, lipid profile and inflammation variables. Statistical significance was accepted at the 0.05 level.

## 3. Results

### 3.1. Body Composition and Daily PA and HRPF

The changes in body composition and daily PA and HRPF are shown in Table 3. Body weight (*p* = 0.001), BMI (*p* = 0.001), LBM (*p* = 0.023), percent body fat (*p* = 0.000), WC (*p* = 0.000), HC (*p* = 0.000), MVPA (*p* = 0.000), step count (*p* = 0.000), sedentary behavior (*p* = 0.000), hand grip strength (*p* = 0.014), repeated chair stand (*p* = 0.003), TFS (*p* = 0.003), and 2 min walking (*p* = 0.003) show a valid difference in the interaction between the group and time.

### 3.2. CVD Risk Factor and EAT

Table 4 shows the changes in CVD risk factor and EAT. In the TKD group, TC (*p* < 0.001), TG (*p* < 0.01), LDL-C (*p* < 0.01), MCP-1 (*p* < 0.05), TNF-α (*p* < 0.05), sVCAM-1 (*p* < 0.001), sE-selectin (*p* < 0.01), MAP (*p* < 0.01), MVO_2_ rest (*p* < 0.05), SBP (*p* < 0.01), DBP (*p* < 0.01), and EAT (*p* < 0.01) decreased validly, and HDL-C (*p* < 0.001) and IL-1β (*p* < 0.01) increased validly. In addition, there was a valid difference in the interaction between the group and time in TC (*p* = 0.002), TG (*p* = 0.008), LDL-C (*p* = 0.000), HDL-C (*p* = 0.001), IL-1β (*p* = 0.001), TNF-α (*p* = 0.042), MCP-1 (*p* = 0.009), sVCAM-1 (*p* = 0.009), sE-selectin (*p* = 0.018), MAP (*p* = 0.000), MVO_2_ rest (*p* = 0.002), SBP (*p* = 0.002), DBP (*p* = 0.000), EAT (*p* = 0.041).

### 3.3. Correlations

The correlations between EAT and CVD risk factor, body composition, and daily PA are shown in Table 5. EAT and IL-1β (r = 0.530, *p* < 0.05), MCP-1 (r = 0.524, *p* < 0.05), TG (r = 0.493, *p* < 0.05), and sedentary behavior (r = 0.459, *p* < 0.05) presented a positive correlation, while EAT and LBM (r = −0.453, *p* < 0.05) showed a negative correlation.

## 4. Discussion

Improvement in the body composition of an elderly person would be more significant in terms of health compared to other ages [48], and the implementation of a TKD program for 12 weeks rendered significant differences in an interaction between groups and time with regard to body weight, BMI, LBM, percent body fat, WC, and HC, suggesting that most of the indicators improved.

Such results correspond with the results of Jeong et al. [49] that reported the improvement in body composition of obese elderly women from long-term TKD aerobic and thera-band exercise. TKD comprises diverse basic movement such as step, jump, punch, and kick, etc., using hands and feet [31], including various physical motions. It can be applicable to the group exercise of an elderly person, and it can not only fulfill the recommended extent of daily PA by ASCM [50] but also is effective in the enhancement of muscular strength [32]. Thus, the TKD program is estimated as a means of effective exercise for elderly women by taking into account the high relevance of TKD, as a combination of aerobic and anaerobic exercise, to body composition, BP, and CVD [43,51,52].

The improvement of the physical strength of an elderly person is important in that a decrease in physical strength would result in reduced social activity in daily life, leaving negative effects comprising increased death rate, reduced activity of daily living, and quality of life (QOL), etc. [53]. The result of the implementation of a TKD program that lasted for 12 weeks rendered significant improvement in interaction between groups and time of hand grip strength, repeated chair stand, TFS, and 2 min walking.

In particular, hand grip strength has been known as an important indicator representing health and disease [54] together with close relevance to mortality rate [55], which was estimated to be significant and revealed the positive effect of improvement. Besides, the development of a diverse exercise program for an elderly person seems necessary by grafting TKD, the Korean martial arts, on physical exercise programs requiring no special tools, which are easily implemented considering prior research [56] that reported a decrease in the level of daily activity by 15% after the age of 60 years and over twice that after 70 years despite differences in the lifestyles of individuals.

Meanwhile, the increase in daily PA has been known to have a positive effect on CVD risk factor [57], and the increase in PA has an effect similar to that of regular exercise [43]. The sedentary lifestyle of an elderly person would not satisfy the minimum amount of PA required to secure their health or constitute regular exercise; thus, such sedentary behavior is classified as a risk factor of CVD and chronic diseases, etc. [43]. Park et al. [1,44] suggested that an elderly person needs an MVPA of over 7–8000 step/day and 15–20 min/day to physical health and to improve geriatric diseases.

The implementation of a TKD program that lasted for 12 weeks rendered significant improvement in MVPA, step count, and sedentary behavior. Such results correspond to the results of previous studies and are considered to be the effects of regular long-term exercise that increased PA through the TKD program.

Besides the increase in PA, which was significant, the decrease in sedentary behavior due to the TKD program was also a very significant result by taking the negative effect of sedentary life over elderly people into account. Therefore, the development of an exercise program designed to reduce the sedentary behavior of an elderly person seems necessary as well as the short-term regular PA or exercise habit.

Globally, hypertension has been regarded as an important issue in the discipline of public health [58]. In particular, the prevalence rate of hypertension in Asia appears higher than that of heart disease and stroke [59]. In addition, Sironi et al. [60] reported the relevance of the accumulation of ectopic fat to essential hypertension, which was the characteristic thereof.

American Heart Association(AHA) [2] emphasized the importance of the control of BP through exercise, and exercise was reported as more positive than drug treatment for the improvement of BP [61]. The group took a TKD program for 12 weeks and showed improvement in SBP (*p* = 0.002) and DBP (*p* = 0.000), and manifested significant differences in terms of the interaction between groups and time of MAP (*p* = 0.000) and MVO_2_ rest (*p* = 0.002). Such results are attributed to the fact that regular exercise intervention effectuated positively over the improvement of BP and reduction in sedentary behavior. In particular, the decrease in DBP by below 90 mmHg through TKD was identified as very effective in the prevention of CVD considering the results of previous studies [62]; thus, the improvement in BP through TKD seems important for the prevention of CVD and improvement of QOL in that hypertension is an important direct risk factor of CVD, the important cause of death, and of QOL that decreases the level thereof [63].

Meanwhile, diverse factors causing CVD such as hypertension, obesity due to absence of PA, and gender, etc., are reported [2,7]. Among them, the EAT, of which characteristic location is concerned, is pointed out as an independent risk factor of CVD [15,16,17] and is employed as an indicator predicting CVD [12,13,14].

Recently, diverse studies examining the effect of intervention through exercises to improve EAT and CVD have been carried out. Manuel et al. [64] reported significant improvement in EAT upon execution of aerobic exercise for adults with metabolic syndrome, wherein the EAT and the risk of CVD appeared to decrease due to PA.

Kalyana et al. [65] mentioned that the implementation of aerobic exercise was the non-pharmacological treatment method that rendered a positive effect over the decrease in the EAT of overweight or obese adult males, and Park et al. [28] reported the high correlation between body composition and CVD risk factor as well as the decrease in EAT thickness upon implementation of swimming exercise for obese adult males.

As shown in the results of such previous studies, the execution of physical exercise seems to have a positive influence upon the improvement of EAT and CVD risk factors. However, most of the previous studies have employed participants of adult males as subjects of each study; therefore, the studies employing elderly women with hypertension as subject are few. In addition, the methods employed for the intervention of exercise was limited to aerobic exercises; thus, various approaches to each exercise are needed. The EAT appeared to decrease by 5.25% after the implementation of a TKD program and rendered significant differences from the interaction between groups and time (*p* = 0.041). Additionally, the TC (*p* = 0.002), TG (*p* = 0.008), LDL-C (*p* = 0.000), and HDL-C (*p* = 0.001) among risk factors of CVD were also appeared with significant improvement. In the present research, the EAT and TG (r = 0.493, *p* < 0.05) and sedentary behavior (r = 0.459, *p* < 0.05) manifested the significant static correlation to each other that identified the effect of TKD upon decrease in EAT compared to the results of previous research that reported the obvious relationship between increase in sedentary behavior and deposition of heart fat.

EAT is a deposition of visceral fat wherefrom the pre-inflammatory adipokine and chemokines including anti-inflammatory adipokine that contains TNF-α, MCP-1, and adiponectin are secreted [66,67,68]. Secretion of inflammatory adipokine suggests the role of EAT that can cause atherosclerosis [20]; the increase in the level of TNF-α and MCP-1 is correlated with the generation of atherosclerosis [69], where the TNF-α increases the manifestation of adhesion molecule [70]. Palmefors et al. [27] reported that PA decreases the cytokine and TNF-α, while aerobic exercise decreases the VCAM-1 and ICAM-1 in the research that reviewed the PA and exercise.

In the present research, the IL-1β (*p* = 0.001), TNF-α (*p* = 0.042), and MCP-1 (*p* = 0.009), which are inflammatory factors, appeared with significant improvement upon completion of TKD that lasted for 12 weeks. By taking the results of previous studies that reported the EAT with the role as an endocrine organ [16] and as the source of inflammatory cytokine into account [19,20], the significant decrease in EAT suggests the significance of improvement of each variable. Additionally, in the present research, the high correlation between EAT and IL-1β(r = 0.530, *p* < 0.05) and MCP-1 (r = 0.524, *p* < 0.05) appeared.

Sacks et al. [71] reported the direct effect of EAT over coronary artery atherosclerosis through the increase in coronary artery calcium and plaque attributable to the discharge of molecules of bioactivity (IL-6, IL-1β, MCP-1) that resulted from the decrease in secretion of adiponectin.

Osborn et al. [72] reported the manifestation of atherosclerosis from an attachment of sVCAM-1 and sE-Selectin, etc., called adhesion molecules, to the wall of blood vessels and endothelial cells that resulted from the surface of inflammatory cells upon the occurrence of inflammation inside of blood vessels and the walls of blood vessels [73].

Besides, it was reported that the secretion of EAT accompanies the increase in the secretion of endothelial cell adhesion molecules (sE-selectin, VCAM-1, ICAM-1) and the promotion of the adhesion of monocyte endothecium [74]. The group that took the TKD program that lasted for 12 weeks appeared with significant improvement in sVCAM-1 and sE-Selectin. By considering the results of previous research, reported EAT plays the roles of endocrine organs and inflammatory cytokine, and the relevance of EAT to atherosclerosis, the decrease in EAT, appeared with a significant effect on the prevention and improvement of the cardiovascular adhesion molecule.

In this study, it was confirmed that the decrease in EAT was correlated with improvement in sedentary behavior, fat mass, lipid profile and inflammatory factors. These results show that elderly women with hypertension improve HRPF and lipid profile and vascular adhesion molecules positively through long-term regular Taekwondo training, and the association of improving CVD risk factors by controlling blood pressure due to decreased EAT was confirmed.

This study has some limitations. First, as a single-center research, the group of study subjects was small and limited to elderly women only. Second, it cannot be assumed that all changes in the variables observed in this study were caused by increased unique effects of TKD only or PA. Therefore, it is necessary to conduct future research including bigger populations and elderly men.

## 5. Conclusions

The 12-week regular Taekwondo intervention is effective in reducing the thickness of EAT measured by MDCT. The reduction in EAT is associated with an improvement in sedentary behavior, lean mass, lipid profile and inflammation factor. There was also significant improvement in HRPF, BP, and the estimated risk factors of CVD in the intervention group compared to the control group. This result could suggest that individuals with hypertension undergoing regular Taekwondo training would be protected from high BP values and their health risks.

## Figures and Tables

**Table 1:** Baseline characteristics of socio-demographic, physical characteristics and blood pressure.

Variable	Taekwondo (*n* = 10)	Control (*n* = 10)	*p*-Value
**Socio-demographic**			
Age (years)	72.90 ± 5.84	71.90 ± 3.11	0.057
Hypertension duration (years)	11.90 ± 4.33	11.30 ± 4.62	0.676
Hypertension drug duration (years)	10.80 ± 3.79	10.40 ± 4.55	0.396
Job, *n* (%)	0 (0%)	0 (0%)	1.000
Smoking (yes), *n* (%)	0 (0%)	0 (0%)	1.000
Alcohol consumption (yes), *n* (%)	0 (0%)	0 (0%)	1.000
**Physical Characteristics and Blood pressure**			
Body height (m)	1.51 ± 0.03	1.50 ± 0.05	0.491
Body weight (kg)	57.48 ± 5.69	56.91 ± 2.97	0.063
Body mass index (kg/m^2^)	25.32 ± 2.54	25.30 ± 1.68	0.176
Lean body mass (kg)	32.81 ± 3.13	34.71 ± 4.34	0.591
Percent body fat (%)	42.82 ± 3.81	42.57 ± 3.66	0.886
Waist circumference (cm)	92.28 ± 6.61	91.96 ± 4.29	0.231
Hip circumference (cm)	100.72 ± 5.42	99.57 ± 3.40	0.283
Waist hip ratio	0.92 ± 0.03	0.92 ± 0.05	0.204
Systolic blood pressure (mmHg)	150.90 ± 7.99	148.20 ± 7.10	0.815
Diastolic blood pressure (mmHg)	92.20 ± 6.32	91.20 ± 6.51	0.982

Values are means ± standard deviations.

**Table 2:** Taekwondo training program.

Items	Contents			Time (min)
Warm-Up	Shuttle Walking, Sitting Stretching			10
The content of Taekwondo training intervention	1~4 week	Stance—Close, Parallel, Riding, Forward, Forward inflection (5 times) Fist punching—Trunk, Face opposite, Underneath (Both 15 times) Blocking—Underneath, Trunk, Face opposite (Both 15 times) Kick—Front, Roundhouse, Downward, Side (Both 15 times) Taekwonrobic (Seperated action 2 times, With rhythm 2 times) (20 min) Step box—Basic step, Knee up, Kick, Leg curl, Tap (20 min)	HRR; 40~59%	70
	5~12 week	Stance—Close, Parallel, Riding, Forward, Forward inflection (5 times) Fist punching—Trunk, Face opposite, Underneath, Side (Both 15 times) Blocking—Underneath, Trunk, Face opposite (Both 15 times) Kick—Front, Roundhouse, Downward, Side (Both 15 times) Taekwonrobic (Seperated action 2 times, With rhythm 2 time) (20 min) Step box—Basic step, Knee up, Kick, Leg curl, Tap (20 min)	HRR; 60~75%	
Cool-down	Shuttle walking, Standing stretching			10
	Jeong et al. (2018, modified program)			

**Table 3:** The changes of body composition and daily physical activity and health-related physical fitness between the groups at baseline and after 12 weeks.

Variable	Group	Baseline	12 Weeks	% Diff	*p*-Value (Interaction)
Body weight (kg)	Taekwondo	57.48 ± 5.69	56.53 ± 5.58	−1.65 ***	0.001
	Control	56.91 ± 2.97	57.21 ± 2.95	0.53	
Body mass index (kg/m^2^)	Taekwondo	25.32 ± 2.54	24.91 ± 2.57	−1.63 ***	0.001
	Control	25.30 ± 1.68	25.44 ± 1.67	0.53	
Lean body mass (kg)	Taekwondo	32.81 ± 3.13	34.60 ± 2.94	5.46 ***	0.023
	Control	34.71 ± 4.34	34.21 ± 4.05	−1.44	
Percent body fat (%)	Taekwondo	42.82 ± 3.81	39.97 ± 4.15	−6.66 ***	0.000
	Control	42.57 ± 3.66	43.91 ± 3.44	3.14	
Waist circumference (cm)	Taekwondo	92.28 ± 6.61	88.36 ± 6.36	−4.25 ***	0.000
	Control	91.96 ± 4.29	93.38 ± 4.68	1.54 *	
Hip circumference (cm)	Taekwondo	100.72 ± 5.42	97.19 ± 5.31	−3.50 ***	0.000
	Control	99.57 ± 3.40	100.26 ± 2.98	0.69*	
Waist hip ratio	Taekwondo	0.92 ± 0.03	0.91 ± 0.02	−0.78	0.080
	Control	0.92 ± 0.05	0.93 ± 0.05	0.82	
Moderate to vigorous physical activity (min/day)	Taekwondo	11.20 ± 8.39	39.90 ± 10.21	256.25 ***	0.000
	Control	12.90 ± 9.84	12.60 ± 8.37	−2.33	
Step count (steps/day)	Taekwondo	3739.90 ± 1220.41	9125.30 ± 2367.07	144.00 ***	0.000
	Control	3361.60 ± 1460.99	3277.00 ± 1418.76	−2.52	
Sedentary behavior (min/day)	Taekwondo	1428.80 ± 8.39	1100.10 ± 10.21	−23.01 ***	0.000
	Control	1427.10 ± 9.84	1427.40 ± 8.37	0.02	
Hand grip strength (kg) (dominant)	Taekwondo	20.93 ± 3.67	23.72 ± 3.94	13.33 *	0.014
	Control	21.68 ± 3.37	20.25 ± 3.99	−6.60	
Repeated chair stand (frequency)	Taekwondo	8.80 ± 1.97	7.34 ± 1.02	−16.57 **	0.003
	Control	8.89 ± 1.43	9.23 ± 1.46	3.85	
Trunk flexion in sitting position (cm)	Taekwondo	14.45 ± 6.02	17.65 ± 5.69	22.15 ***	0.003
	Control	12.78 ± 6.76	13.55 ± 6.63	6.03	
2 min walking (frequency)	Taekwondo	108.40 ± 10.62	118.60 ± 11.60	10.20 ***	0.003
	Control	110.10 ± 19.99	101.90 ± 24.70	−7.45	

Values are means ± standard deviations. Significantly different from baseline compared to 12 weeks: * *p* < 0.05, ** *p* < 0.01, *** *p* < 0.001.

**Table 4:** The changes of cardiovascular disease risk factor and epicardial adipose tissue between the groups at baseline and after 12 weeks.

Variable	Group	Baseline	12 Weeks	% Diff	*p*-Value (Interaction)
Total cholesterol (mg/dL)	Taekwondo	232.10 ± 37.45	203.20 ± 25.44	−12.45 ***	0.002
	Control	231.60 ± 29.28	232.30 ± 27.85	0.30	
Triglyceride (mg/dL)	Taekwondo	171.10 ± 16.50	154.90 ± 15.72	−9.47 **	0.008
	Control	170.20 ± 18.19	169.50 ± 20.45	−0.41	
Low density lipoprotein cholesterol (mg/dL)	Taekwondo	119.30 ± 22.93	100.50 ± 18.04	−15.76 **	0.000
	Control	118.50 ± 13.11	121.50 ± 12.39	8.01	
High density lipoprotein cholesterol (mg/dL)	Taekwondo	49.40 ± 7.66	59.50 ± 9.00	20.45 ***	0.001
	Control	50.50 ± 8.89	49.50 ± 9.07	−1.98	
Monocyte chemotactic protein-1 (pg/mL)	Taekwondo	447.59 ± 52.64	407.30 ± 67.12	−9.00 *	0.009
	Control	454.87 ± 69.89	474.79 ± 82.54	4.38	
Soluble vascular cell adhesion molecule-1 (ng/mL)	Taekwondo	802.61 ± 62.44	739.02 ± 85.16	−7.90 ***	0.009
	Control	837.37 ± 105.90	855.72 ± 111.33	2.19	
Soluble intercelluar cell adhesion molecule-1 (ng/mL)	Taekwondo	210.02 ± 73.96	189.16 ± 62.39	−9.93	0.181
	Control	211.71 ± 68.96	254.99 ± 62.26	20.44	
Soluble E-selectin (ng/mL)	Taekwondo	49.76 ± 18.71	40.79 ± 15.90	−18.02 **	0.018
	Control	48.24 ± 14.10	48.50 ± 15.23	0.56	
Interukin-1β (pg/mL)	Taekwondo	0.16 ± 0.04	0.12 ± 0.04	−21.02 **	0.001
	Control	0.14 ± 0.02	0.16 ± 0.02	11.35	
Tumor necrosis factor-α (pg/mL)	Taekwondo	2.30 ± 1.08	1.34 ± 0.58	−48.64 *	0.042
	Control	2.25 ± 1.60	2.66 ± 1.71	18.12	
High-sensitivity C-reactive protein (mg/L)	Taekwondo	0.81 ± 0.69	0.63 ± 0.32	−21.05	0.069
	Control	0.79 ± 0.72	1.41 ± 0.73	43.92 **	
Pulse pressure (mmHg)	Taekwondo	58.70 ± 3.77	61.00 ± 12.42	3.92	0.664
	Control	57.00 ± 3.50	57.50 ± 4.70	0.88	
Mean arterial pressure (mmHg)	Taekwondo	111.77 ± 6.69	96.23 ± 10.96	−13.90 **	0.000
	Control	110.20 ± 6.51	112.47 ± 4.44	2.06	
MVO_2_ rest (mmHg × beats/min × 10^−3^)	Taekwondo	11.52 ± 0.83	10.45 ± 1.10	−9.28 *	0.002
	Control	11.31 ± 0.66	11.52 ± 0.73	1.82	
Systolic blood pressure (mmHg)	Taekwondo	150.90 ± 7.99	136.90 ± 12.46	−9.28 **	0.002
	Control	148.20 ± 7.10	150.80 ± 5.80	1.75 **	
Diastolic blood pressure (mmHg)	Taekwondo	92.20 ± 6.32	75.90 ± 12.40	−17.68 **	0.000
	Control	91.20 ± 6.51	93.30 ± 4.50	2.30	
Epicardial adipose tissue (mm^2^)	Taekwondo	1995.20 ± 911.79	1890.50 ± 893.16	−5.25 **	0.041
	Control	1541.80 ± 367.67	1609.30 ± 438.39	4.38	

Values are means ± standard deviations. Significantly different from baseline compared to 12 weeks: * *p* < 0.05, ** *p* < 0.01, *** *p* < 0.001.

**Table 5:** Correlation coefficient between epicardial adipose tissue and cardiovascular risk factor (*n* = 20).

Variable	Epicardial Adipose Tissue (mm^2^)
Lean body mass (kg)	−0.453 *
Sedentary time (min/day)	0.459 *
Triglyceride (mg/dL)	0.493 *
Monocyte chemotactic protein-1 (pg/mL)	0.524 *
Interukin-1β (pg/mL)	0.530 *

Analyzed by Pearson’s correlation coefficients, * *p* < 0.05.

## Data Availability

Qualified researchers can obtain the data from the corresponding author (htpark@dau.ac.kr and sgpark@dau.ac.kr). The data are not publicly available due to privacy concerns imposed by the IRB ethical principles.

## References

[1] Hardin E.A., Chin K.M. (2016). Selexipag in the treatment of pulmonary arterial hypertension: Design, development, and therapy. Drug Des. Dev. Ther..

[2] American Heart Association (2015). Heart disease and stroke statistics–2015 update: A report from the American Heart Association. Circulation.

[3] Statistics Korea (2018). 2018 Elderly Person Statistics.

[4] Dam R.M.T., Spiegelman D., Franco O.H., Hu F.B. (2008). Combined impact of lifestyle factors on mortality: Prospective cohort study in US women. BMJ.

[5] Jia G., Jia Y., Sowers J.R. (2011). Contribution of maladaptive adipose tissue expansion to development of cardiovascular disease. Compr. Physiol..

[6] Skarn S.N., Eggesbo H.B., Flaa A., Kjeldsen S.E., Rostrup M., Brunborg C., Reims H.M., Aksnes T.A. (2016). Predictors of abdominal adipose tissue compartments: 18-years follow-up of young men with and without family history if diabetes. Eur. J. Intern. Med..

[7] Chan A.W.K., Chair S.Y., Lee D.T.F., Leung D.Y.P., Sit J.W.H., Cheng H.Y., Taylor-Piliae R.E. (2018). Tai Chi exercise is more effective than brisk walking in reducing cardiovascular disease risk factors among adults with hypertension: A randomised controlled trial. Int. J. Nurs. Stud..

[8] Manson J.E., Willett W.C., Stampfer M.J., Colditz G.A., Hunter D.J., Hankinson S.E., Hennekens C.H., Speizer F.E. (1995). Body weight and mortality among women. N. Engl. J. Med..

[9] Albu J.B., Kovera A.J., Johnson J.A. (2000). Fat distribution and health in obesity. Ann. N. Y. Acad. Sci..

[10] Wilson P.W.F., D’Agostino R.B., Sullivan L., Parise H., Kannel W.B. (2002). Overweight and obesity as determinants of cardiovascular risk: The Framinham experience. Arch. Intern. Med..

[11] Taguchi R., Takasu J., Itani Y., Yamamoto R., Yokohama K., Watanabe S., Masuda Y. (2001). Pericardial fat accumulation in men as a risk factor for coronary artery disease. Atherosclerosis.

[12] Iacobellis G., Pistilli D., Gucciardo M., Leonetti F., Miralfi F., Brancaccio G., Gallo P., Di Gioia C.R.T. (2005). Adiponecting expression in human epicardail adipose tissue in vivo is lower in patients with coronary artery disease. Cytokine.

[13] Iacobellis G., Barbaro G. (2008). The double Role of Epicardial Adipose Tissue as pro-and Anti-inflammatory organ. Horm. Metab. Res..

[14] Icobellis G., Ribaudo M.C., Assael F., Vecci E., Tiberti C., Zappaterreno A., Di Mario U., Leonetti F. (2003). Echocardiographic epicardial adipose tissue is related to anteropometric and clinical parameters of metabolic syndrome: A new indicator of cardiovascular risk. JCEM.

[15] Ra J.S., Kim H.S. (2015). Comined Influence of Obesity and Metabolic Syncrome on Ischemic Heart Disease in Korean middle aged and older adults. J. Korean Public Health Nurs..

[16] Hikmet Y., Ugur C., Tuncay H., Ahmet H.A., Hamza S., Muhammet D., Levent S., Ergun B.K., Kudret A., Lale T. (2011). Increased epicardial fat tissue is a marker of metabolic syndorme in adult patients. Int. J. Cardiol..

[17] Yang C., Li L., Zha Y., Peng Z. (2016). Correlation between epicardial adipose tissue and severity of coronary artery stenosis evulated by 64-MDCT. Clin. Imaging.

[18] Gonzalez N., Moreno-Villegas Z., Gonzalez-Bris A., Egido J., Lorenzo O. (2017). Regulation of visceral and epicardial adipose tissue for preventing cardiovascular injuries associated to obesity and diabetes. Cardiovasc. Diabetol..

[19] Iozzo P. (2011). Myocardial, perivascular, and epicardial fat. Diabetes Care Suppl..

[20] Mazurek T., Zhang L., Zalewski A. (2003). Human epicardial adipose tissue is a source of inflammatory mediator. Circulation.

[21] Eszter N., Adam L.J., Bela M., Pal M.H. (2017). Clinical importance of epicardial adipose tissue. Arch. Med. Sci..

[22] Ferrari A.U., Radaelli A., Centola M. (2003). Physiology of aging. Invited review: Aging and the cardiovascular system. J. Appl. Physiol..

[23] Larsen B.A., Allison M.A., Kang E., Saad S., Laughlin G.A., Araneta M.R.G., Barrett-Connor E., Wassel C.L. (2014). Associations of Physical Activity and Sedentary Behavior with Regional Fat Deposition. Med. Sci. Sports Exerc..

[24] Kim M.K., Tanaka K., Kim M.J., Matsuo T., Tomita T., Ohkubo H., Maeda S., Ajisaka R. (2010). Epicardial fat tissue: Relationship with cardiorespiratory fitness in men. Med. Sci. Sports Exerc..

[25] Eijsvogels T.M.H., Molossi S., Lee D.J., Emery M.S., Thompson P.D. (2016). Exercise at the extremes: The amount of exercise to reduce cardiovascular events. J. Am. Coll. Cardiol..

[26] American Heart Association Cardiovascular Disease & Diabetes. Updated 2018. https://www.heart.org/en/health-topics/diabetes/why-diabetes-matters/cardiovascular-disease--diabetes.

[27] Palmefors H., DuttaRoy S., Rundqvist B., Borjesson M. (2014). The effect of physical activity or exercise on key biomarkers in atherosclerosis—A systematic review. Atherosclerosis.

[28] Park S.H., So Y.S., Kim M.J., Kim D.H., Yoon M.Y., Kim Y.J. (2015). Effect of Swimming Exercise on Epicardial Fat and Cardiovascular Risk Factor in Obese Adult Men. J. Coach. Dev..

[29] Kim M.K., Tomita T., Kim M.J., Sasai H., Maeda S., Tanaka K. (2009). Aerobic exercise training reducese picardial fat in obese men. J. Appl. Physiol..

[30] Roh H.T., Cho S.Y., So W.Y. (2018). Taekwondo Training Improves Mood and Sociability in Children from Multicultural Families in South Korea: A Randomized Controlled Pilot Study. Int. J. Environ. Res. Public Health.

[31] Bridge C.A., Da Silva Santos J.F., Chaabène H., Franchini E. (2014). Physical and physiological profiles of taekwondo athletes. Sports Med..

[32] Lee S.H., Park S.K., Hong G.R. (2018). The effect of taekwondo training on physical fitness and the allergic response factor of hypersensitive obese children. Arch. Budo.

[33] Bridge C.A., Jones M.A., Hitchen P., Sanchez X. (2007). Heart rate responses to Taekwondo training in experienced practitioners. J. Strength Cond. Res..

[34] Lee Y.H., Kim K.T. (2013). Tai Chi as a Fall Prevention Intervention: An In-depth Literature Review of Randomized Controlled Trials and Suggestion to Taekwondo. Off. J. Korean Acad. Kinesiol..

[35] Jeong M.K., Jung H.H. (2020). Effects of Silver Taekwondo Program on Self-reliance Physical Fitness, Depressive Symptoms and Diabetes Factors in Obese Elderly Women with Type 2 Diabetes. Korea J. Sports Sci..

[36] American College of Sports Medicine (2006). ACSM’s Guidelines for Exercise Testing and Prescription.

[37] Gobel F.L., Norstrom L.A., Nelson R.R., Jorgensen C.R., Wang Y. (1978). The rate-pressure product as an index of myocardial oxygen consumption during exercise in patients with angina pectoris. Circulation.

[38] Park H., Park J.H., Na H.R., Shimada H., Kim G.M., Jung M.K., Kim W.K., Park K.W. (2019). Combined Intervention of Physical Activity, Aerobic Exercise, and Cognitive Exercise Intervention to Prevent Cognitive Decline for Patients with Mild Cognitive Impairment: A Randomized Controlled Clinical Study. J. Clin. Med..

[39] Jeong M.K., Lee S.H., Ryu J.K., Kim Y.H., Kim E.H., Hong G.R., Park J.H., Baek S.H., Park S.K., Jung H.H. (2019). Effects of long-term multi-task exercise program on blood pressure, physical function and cognitive function in mild cognitive impairment elderly women with hypertension. Arch Budo.

[40] Park H., Togo F., Watanabe E., Yasunaga A., Park S., Shephard R.J., Aoyagi Y. (2007). Relationship of bone health to yearlong physical activity in older Japanese adults: Cross-sectional data from the Nakanojo Study. Osteoporos. Int..

[41] Park H., Park S., Shephard R.J., Aoyagi Y. (2010). Yearlong physical activity and sarcopenia in older adults: The Nakanojo Study. Eur. J. Appl. Physiol..

[42] Park J.H., Park S.K., Lee D.M., Jeong M.K., Kim D.H., Lee T.H., Jeon S.H., Park J.K., Kim E.H., Min S.K. (2014). The Relationship between Sedentary Behavior and Cystatin C, Blood Pressure in Obese Elderly Women. Korea J. Sports Sci..

[43] American College of Sports Medicine (2018). ACSM’s Guidelines for Exercise Testing and Prescription.

[44] Gaber C.E., Blissmer B., Deschenes M.R., Franklin B.A., Lamonte M.J., Lee I.M., Nieman D.C., Swain D.P., ACSM (2011). American College of Sports Medicine position stand. Quantity and quality of exercise for developing and maintaintaining cardiorespiratory, musculoskeletal, and neuromotor fitness in apparently healthy adults: Guidance for prescribing exercise. Med. Sci. Sports Exerc..

[45] Pescatello L.S., MacDonald H.V., Ash G.I., Lamberti L.M., Farquhar W.B., Arena R., Johnson B.T. (2015). Assessing the Existing Professional Exercise Recommendations for Hypertension: A Review and Recommendations for Future Research Priorities. Mayo Clin. Proc..

[46] Eckel R.H., Jakicic J.M., Ard J.D., De Sesus J.M., Miller N.H., Hubbard V.S., Lee I.M., Lichtenstein A.H., Loria C.M., Millen B.E. (2014). 2013 AHA/ACC guideline on lifestyle management to reduce cardiovascular risk: A report of the American College of Cardiology/American Heart Association Task Force on Practice Guidelines. J. Am. Coll. Cardiol..

[47] Jeong M.K., Ryu J.K., Jung H.H., Kim H.W., Park S.K. (2018). Effects of Taekwondo aerobic and Combined Exercise Program on Health-related Physical Fitness and Physical Activity and Depression Scale in Menopausal Obesity Women. Korea J. Sports Sci..

[48] Kim E.H., Park S.K., Hong G.R. (2019). Effects of TTM and Longevity Exercise Program on Health-related Physical Fitness, Blood Pressure and IMT in Elderly Women with Hypertension. Korean Soc. Sports Leis. Stud..

[49] Jeong M.K., Park H.T., Park S.K., Kim E.H., Kwon Y.C. (2015). Effects of Long-term Taekwondo Aerobic and Thera-band Training on Self-reliance Physcal Fitness, and hs-CRP concentration in Obese Elderly Women. Korea J. Sports Sci..

[50] Toskovic N.N., Blessing D., Williford H.N. (2002). The effect of experience and gender on cardiovascular metabolic responses with dynamic Tae Kwon Do exercise. J. Strength. Cond. Res..

[51] Stewart K.J., Bacher A.C., Turner K.L., Fleg J.L., Hees P.S., Shapiro E.P., Tayback M., Ouyang P. (2005). Effect of exercise on blood pressure in older persons: A randomized controlled trial. Archives Intern. Med..

[52] Kim E.H., Park S.K., Kim E.Y., Hong G.R. (2013). Effects of Combined Exercise Program on Glucose, Cardiovascular Disease Risk Factors and Health-Related Quality of Life in Elderly Women with Type II Diabetes. Korea J. Sports Sci..

[53] Ministry of Culture, Sports and Tourism (2017). 2017 Korean National Health Status Survey.

[54] Bohannon R.W., Bear-Lehman J., Derosiers J., Massy-westropp N., Mathiowets V. (2015). Average grip strength: A meta-analysis of data obtained with a Jamar Dynamometer from individuals 75 years or more of age. J. Geriatr. Phys. Ther..

[55] Rijk J.M., Roos P.R., Deckx L., Van den Akker M., Buntinx F. (2016). Prognostic value of handgrip strength in people aged 60 years and older: A systematic review and meta-analysis. Med. Index..

[56] Rogers M.A., Evans W.J. (1993). Changes on skeletal muscle with aging. effects of exercise training. Exerc. Sports Sci. Rev..

[57] Pescatello L.S., Vanheest J.L. (2000). Physical activity mediates a healthier body weight in the presence of obesity. Br. J. Sports Med..

[58] Keith M.D., Daichi S. (2013). Physical Activity and the Prevention of Hypertension. Curr. Hypertens. Rep..

[59] Cheng H.M., Chiang C.E., Chen C.H. (2015). The Novelty of the 2015 Guidelines of the Taiwan Society of Cardiology and the Taiwan Hypertension Society for the Management of Hypertension. Pulse.

[60] Sironi A.M., Gastaldelli A., Mari A., Ciociaro D., Postano V., Buzziggoli E., Ghione S., Turchi S., Lombardi M., Ferrannini E. (2004). Visceral fat in hypertension: Influence on insulin resistance and beta-cell function. Hypertension.

[61] Adriana L.S., Luciana M.L., Eliane A.C., Renato P.V., Wellongton S., Thaos C.Z., Leon A.D. (2015). Blood pressure in hypertensive women after aerobics and hydrogymnastics sessions. Nutr. Hosp..

[62] Stanley S.F., Nathan D.W. (2013). Hypertension and Cardiovascular Disease: Contributions of the Framingham Heart Study. Glob. Heart.

[63] Korea Centers for Disease Control and Prevention (2017). The Seventh Korea National Health and Nutrition Examination Survey (KNHANES VII-2).

[64] Manuel M., Paolo D.R., Maria R.P., Francesco B., Giovanni M.V. (2015). New evidences about the strict relationship between the epicardial fat and the aerobic exercise. IJC Metab. Endocr..

[65] Kalyana C.B., Arun G.M., Padma K., Krishnananda N., Vasudeva G., Vidya N. (2018). Effect of aerobic exercise on echocardiographic epicardial adipose tissue thickness in overweight individuals. Diabetes Metab. Syndr. Obes..

[66] Talman A.H., Psaltis P.J., Cameron J.D., Meredith I.T., Senevirantne S.K., Wong D.T.L. (2014). Epicardial adipose tissue: Far more than a fat depot. Cardiovasc. Diagn. Ther..

[67] Iacobellis G. (2015). Local and systemic effects of the multifaceted epicardial adipose tissue depot. Nat. Rev. Endocrinol..

[68] Gunasekar P., Swier V.J., Fleegel J.P., Boosani C.S., Radwan M.M., Agrawal D.K. (2018). Vitamin D and Macrophage Polarization in Epicardial Adipose Tissue of Atherosclerotic Swine. PLoS ONE.

[69] Libby P., Ridker P.M., Maseri A. (2002). Inflammation and atherosclerosis. Circulation.

[70] Zhang H., Zhang C. (2012). Vasoprotection by dietary supplements and exercise: Role of TNFalpha signaling. Exp. Diabetes. Res..

[71] Sacks H.S., Fain J.N. (2007). Human epicardial adipose tissue: A review. Am. Heart J..

[72] Osborn L., Hession C., Tizard R., Vassallo C., Luhowskyj S., Chi-Rosso G., Lobb R. (1989). Direct expression cloning of vascular cell adhesion molecule 1, a cytokine-induced endothelial protein that binds to lymphocytes. Cell.

[73] Gearing A.J.H., Hemingway I., Pigoit R., Hughes J., Rees A.J., Cashman S.J. (1992). Soluble forms of vascular adhesion molecules, E-selectin, ICAM-1, and VCAM-1: Pathological significance. Ann. N. Y. Acad. Sci..

[74] Dimitriadis G.K., Kaur J., Adya R., Miras A.D., Mattu H.S., Hattersley J.G., Kaltsas G., Tan B.K., Randeva H.S. (2018). Chemerin induces endothelial cell inflammation: Activation of nuclear factor-kappa beta and monocyte-endothelial adhesion. Oncotarget.

